# Glyceryl trinitrate therapy for ischaemia, painful diabetic neuropathy, healing of foot ulceration and other podiatric conditions: a literature review

**DOI:** 10.1186/1757-1146-4-S1-O28

**Published:** 2011-05-20

**Authors:** Sylvia McAra

**Affiliations:** 1 School of Community Health, Podiatry Department, Charles Sturt University, Albury, NSW, 2640, Australia

## 

Glyceryl trinitrate is a substance that causes vasodilation by donation of nitric oxide which causes relaxation of the endothelium of blood vessel walls. The vasodilatory effect of this substance has been known since 1870 and it has been used extensively in the treatment of angina via transdermal patches, sublingual tablets and sprays. Studies involving normal vasculature as well as diseased vascular states have been promising in terms of demonstrably consistent and significant vasodilatory effects on both systemic and peripheral systems. Podiatric application for this pharmacologic intervention seems to have received little attention in the literature to date. Its efficacy in equine treatment of laminitis, an ischaemic condition of the horses hoof has been well established and researched. It has been applied to other clinical problems involving perfusion of the extremities, such as treatment of inability to achieve penile erection and use in treatment of anal fissures. A literature search has produced information on this drug which suggests potential for greater application in clinical podiatry. This includes positive effects in digital perfusion, which holds promise for wound healing and shows potential for reducing amputations associated with peripheral vascular disease (PVD). This novel therapy has the potential to be useful in cases with borderline vascular supply that require a boost to perfusion to trigger the healing process and/-or relieve other symptoms of ischaemia. It has also been found to be of assistance in the management of painful diabetic neuropathy (PND). This finding is of particular interest as an adjunct to treatment for the prevalent and difficult clinical challenge of PND. The literature regarding its use in musculotendinous applications such as for Achilles tendinopathy will also be covered in this review. Potential issues with this drug including side effects and tolerance will be addressed. Further study of this treatment modality would be beneficial in terms of determining specific indications and limitations of its use, particularly for vascular insufficiency of the feet and healing of foot ulceration.

